# How Do Mothers Living in Socially Deprived Communities Perceive Oral Health of Young Children? A Qualitative Study

**DOI:** 10.3390/ijerph18073521

**Published:** 2021-03-29

**Authors:** Amit Arora, Dimitri Lucas, Michael To, Ritesh Chimoriya, Sameer Bhole, Santosh Kumar Tadakamadla, James J. Crall

**Affiliations:** 1 School of Health Sciences, Western Sydney University, Locked Bag 1797, Penrith, NSW 2751, Australia; r.chimoriya@westernsydney.edu.au; 2 Translational Health Research Institute, Western Sydney University, Locked Bag 1797, Penrith, NSW 2751, Australia; 3 Discipline of Child and Adolescent Health, Sydney Medical School, Faculty of Medicine and Health, Westmead, NSW 2145, Australia; 4 Oral Health Services, Sydney Local Health District and Sydney Dental Hospital, NSW Health, Surry Hills, NSW 2010, Australia; sameer.bhole@health.nsw.gov.au; 5 Sydney Dental School, Faculty of Medicine and Health, The University of Sydney, Surry Hills, NSW 2010, Australia; thetlcdentist@gmail.com (D.L.); michaelto@live.com.au (M.T.); 6 School of Medicine, Western Sydney University, Campbelltown, NSW 2560, Australia; 7 School of Dentistry and Oral Health, Griffith University, Gold Coast, QLD 4222, Australia; santoshkumar.tadakamadla@griffithuni.edu.au; 8 Division of Public Health and Community Dentistry, School of Dentistry, University of California Los Angeles, Los Angeles, CA 90095, USA; jcrall@dentistry.ucla.edu

**Keywords:** oral health, early childhood, qualitative study, prevention, social determinants

## Abstract

This qualitative study aims to explore and gain an in-depth understanding of the knowledge and perceptions of mothers living in Greater Western Sydney (GWS), one of Australia’s most socio-economically disadvantaged regions, regarding the factors that influence oral health of young children. Mother–child dyads (*n* = 45) were purposively selected from a population-based cohort study in GWS. Semi-structured in-depth interviews were audio-recorded, transcribed verbatim, and subsequently analyzed using thematic analysis. Five main themes emerged from the interviews: (1) beliefs about child oral health and first set of teeth; (2) awareness and attitudes towards oral health services; (3) identification of caries risk and protective factors; (4) broader cultural and social class influences on childhood oral health practices; and (5) the influence of parental self-confidence, self-efficacy, and perceived control. Overall, mothers reported having limited knowledge and awareness on the importance of baby teeth, child’s first dental visit, and seeking oral health care. Oral health and preventative practices in children were reported to be influenced by past dental experiences, culture and social class, and parental factors. The empirical findings of this study bring our attention to the critical factors that influence child oral health and the opportunities for co-creating child oral health promotion by targeting mothers.

## 1. Introduction

Despite an overall decline in caries experience in populations as a whole, dental caries continues to be a significant international public health issue [1]. Dental caries is one of the most prevalent preventable diseases, with a reported prevalence of over 90% in children aged 3 to 5 years in some countries [2]. In the Australian context, the most recent National Child Oral Health Survey 2012–2014 reported that nearly 25% of children aged 5 to 10 years had untreated caries in the primary (baby) teeth, while one in ten children aged 6 to 14 years had untreated caries in the permanent dentition [3]. Untreated dental caries may result in negative ramifications, such as discomfort, swelling, severe pain, poor nutrition, loss of sleep, diminished growth and development, and reduction in children’s quality of life [4,5,6]. Past caries experience in the primary dentition is one of the strongest predictors for caries experience in the permanent dentition [7,8]. Therefore, it is important to adopt measures to prevent the development and progression of dental caries at an early age.

Whilst dental caries has a predominant bacteriological aetiology, it is a multi-factorial disease influenced by various determinants. These include poor health behaviors, such as frequent sugar consumption and infrequent toothbrushing with a fluoride toothpaste, low health literacy, poor dental care utilization or lack of access to oral health care, low socio-economic status, and cultural beliefs and practices [9,10,11]. Research suggests that a family-based approach may be an effective solution for dental caries prevention, where families are provided with oral health education to ensure early adoption of healthy behaviors [12,13]. The health promotion messages for good oral health are well-known and include twice daily toothbrushing with a fluoride toothpaste, reducing the frequency of sugar consumption, and seeking visits to an oral health professional on a regular basis [12,13]. However, education and knowledge of good oral health practices alone rarely lead to sustained behavior change as these oral health behaviors are enmeshed in complex daily habits that are influenced by a wide range of psychosocial, environmental, and economic factors [14].

The conceptual model of the influences on children’s oral health by Fisher-Owens et al. [15] has identified the presence of a complex interplay of causal factors. The model clearly depicts the multilevel influences on child’s oral health at individual, family, and community levels, with interactions occurring along various levels of influence. Some of the child-level influences include health behaviors and practices, diet and development, and use of dental care; family-level influences include socio-economic status, social support, health status of parents, culture, and health behaviors, practices, and coping skills of family; and community-level influences include social environment, health and dental care system, culture and community oral health environment [15]. Within the Fisher-Owens model, “health behaviors and practices” is identified as an important determinant of oral health at both the child and family levels [15].

The importance of parental factors on child oral health outcomes is well-established [16,17,18]. Parents and caregivers in particular play a significant role in establishing good oral health practices in their children either directly or indirectly as role models [19]. Numerous studies have reported a significant association between mothers’ oral health and their children’s oral health [20,21]. Mothers primarily play an important role as decision-makers for their child’s overall health and well-being. Mothers’ perception of achieving and maintaining good oral health in terms of instilling positive and healthy behaviors, such as habituation of twice daily toothbrushing with a fluoride toothpaste, control of sugary diet, and seeking regular visits to the oral health professional have been reported as important drivers of their children’s oral health [14]. On the contrary, studies have also reported on the barriers, such as lack of knowledge of proper feeding patterns and cultural beliefs, which could lead to poor oral health outcomes in children [22,23,24]. Research also suggests that social norms, particularly those related to education, ethnicity, and social class strongly influence health-related behaviors [15,25].

Additional research, especially qualitative research exploring the perspectives of parents, is needed to gain a deeper understanding of the factors that influence oral health in early childhood [14,23]. As parents play a vital role in shaping children’s oral health behaviors, recording their perspectives may help guide the development of interventions targeted at improving oral health behaviors [14]. Previous qualitative studies [14,22,24,26] conducted in diverse settings around the world to explore parental views have reported a wide range of determinants of child oral health. However, no qualitative investigation of mother’s perspectives on child oral health has been conducted in the Greater Western Sydney (GWS) region.

The GWS region is one of the fastest growing regions in New South Wales [27], and is currently home to 2.5 million people [28]. Its resident population is ethnically and culturally diverse [29]; and it is one of the most socio-economically disadvantaged regions in Australia [30]. The present study seeks to explore the perspectives of mothers residing in socially deprived communities in particular, as marginalised population groups who experience socio-economic disadvantages and vulnerabilities are often underrepresented in oral health research [31]. Whilst epidemiological evidence [9,11] has identified several factors that hinder and promote oral health in early childhood, there is limited evidence and understanding of what mothers, especially those residing in socio-economically disadvantaged areas and those from culturally and linguistically diverse communities, believe influences early childhood oral health. Identifying those beliefs is paramount to understanding the factors that parents identify as contributing to children’s oral health and to the design of effective interventions to prevent early childhood caries and promote healthy behaviors in early life. The aim of this study was to explore and gain an in-depth understanding of the knowledge and perceptions of mothers living in GWS regarding the factors that influence oral health of young children.

## 2. Methods

### 2.1. Study Background

This qualitative study was part of the larger Healthy Smiles Healthy Kids (HSHK) birth cohort study (*n* = 1035) [32]. Commenced in 2010, the primary aim of the HSHK study was to assess the relationship between early childhood feeding practices, dental caries, and obesity in young children residing in GWS. The rationale and methods of the HSHK study have been described in detail elsewhere [32,33,34].

### 2.2. Ethics Approval

Ethical approval for this study was obtained from the Human Research Ethics Committee of the former Sydney South West Area Health Service- RPAH Zone (ID number X08-0115), Western Sydney University, and the University of Sydney. This research has been conducted in full accordance with the World Medical Association Declaration of Helsinki. Written consent was obtained from all study participants.

### 2.3. Research Design

The qualitative research design employed in this study has been shown to be a valuable approach to explore complex social phenomena and collect in-depth information, particularly in disadvantaged communities [35], on consumers’ and health professionals’ perceptions, understanding and experiences concerning broad issues [14,26,36]. A qualitative approach was used for the following reasons: its exploratory open nature was well-suited to understanding the experiences and perceptions of participants; the flexibility of the study design gave an opportunity for further investigation, if required, and fostered simultaneous data collection and analysis; and it allowed for new perspectives and themes to emerge [36]. Qualitative data also allowed for in-depth exploration of the indirect processes that are primarily involved in the adoption of health behaviors [14]. The interview guides were developed using the Fisher-Owens model [15] to gain an understanding of mothers’ perceptions on the factors that influence child oral health in socially deprived communities.

The qualitative methods and reporting of results in this study adhered to the Consolidated Criteria for Reporting Qualitative Research (COREQ) [37], which is a 32-item checklist used in qualitative studies (Supplementary Table S1).

### 2.4. Sampling

A purposive sampling technique was used to select mother–child dyads for this nested qualitative study. This technique is commonly used in qualitative research, where subjects are selected purposefully and strategically to obtain information-rich data [38]. Maximum variation sampling strategy, which allows capture of varied dimensions of interest and identification of important patterns, was used to further enrich data quality [36,38]. Recruitment of participants continued until the study reached data saturation, the point where all dimensions of interest were explored and no new information seemed to be added from new participants [36].

From the HSHK study (*n* = 1035), a sample of 45 mothers of young children (aged 2–3 years) were selected for a home interview. Mothers were selected from postcodes classified as “disadvantaged” according to the 2016 Socio-Economic Indexes for Areas (SEIFA) developed by the Australian Bureau of Statistics [30]. The SEIFA ranks areas in Australia according to the relative socio-economic advantage and disadvantage. Rankings are provided to areas and not individuals, and indicate the collective socio-economic characteristics of the people living in the area [30].

To ensure a broader perspective, mothers selected for this nested study were:

1. either primiparous or multiparous
2. either married, or living with a partner, or single
3. from a range of education levels
4. either employed (skilled/unskilled) or unemployed and/or pensioners
5. from a diverse ethnic background but able to speak in English

These characteristics were chosen as they are analytically important and have been shown to play an important role in individual and family oral health [39]. Mothers were invited to participate in the study through a telephone call. An information pack containing a participant information statement and a consent form was sent to the participants prior to the interview.

### 2.5. In-Depth Semi-Structured Interviews

In-depth semi-structured interviews were conducted by three researchers (D.L., M.T., and A.A.) between the period October 2017 to December 2018. Face-to-face, one-hour interviews were conducted at the participants’ homes to help establish a rapport with participants and to obtain in-depth responses from a variety of perspectives. A semi-structured interview guide (Table 1) was used to allow for exploration of various related topics of discussion. The development of the interview guide was informed by a comprehensive review of the literature to identify key areas of interest [14,15,26]. The draft interview guide was piloted with four primary caregivers not involved in the HSHK study whose children were 2 to 6 years old. Where appropriate, interviewers probed the participants to discuss any issues outside of the interview schedule. Interviews were continued until no new topics emerged, that is, data saturation was reached. All interviews were audio-recorded, debriefed, and transcribed verbatim using a professional transcription service.

### 2.6. Data Analysis

Thematic analysis was undertaken to interpret the main findings of the interview transcripts [40]. The five steps of thematic analysis followed were as follows: familiarization with the data, generate initial codes, search for themes, reviewing themes, and define and name themes [40]. Some processes were conducted simultaneously, resulting in three stages. First, the principal researcher (A.A.) reviewed all transcripts and used the NVivo 9 (QSR International, Cambridge, MA, USA) software for initial coding and identification of common themes. Second, three researchers (M.T., D.L., and R.C.) independently reviewed the data for manual coding and analysis, and worked through each transcript systematically to identify underlying concepts. Third, all four researchers then reviewed and compared the results of the NVivo coding and the independent manual coding, and where possible, coding was merged to form themes. A thematic map (Supplementary Figure S1) was developed indicating the interaction between themes and subthemes. All the researchers reached a consensus on any discrepant categorization through an ongoing discussion.

### 2.7. Rigor

In order to enhance the rigor of the study, a number of methodological strategies were adopted. Face-to-face interviews were conducted by three researchers with prior experience in qualitative interviewing and population oral health. Interview debriefing between the researchers were consistently undertaken to review completeness of data, summarize the main findings, and determine additional areas to explore in subsequent interviews, which continued until confirmation of data saturation. To ensure accuracy of the verbatim transcriptions of the audio-recordings, a professional transcription service was employed. The interview transcripts were also provided to the participants to check for accuracy. Moreover, four researchers independently checked the data for accuracy and performed the coding, and a team consensus was achieved. Negative case analysis was also conducted to boost rigor. Along with sufficient information about the study setting, participants, and data collection, direct quotes of the participants are presented in the results. With the use of these strategies, the criteria for robust qualitative research including transferability, confirmability, dependability, and credibility have been addressed [41,42].

### 2.8. Researcher Positionality

Researcher positionality as perceived by the study participants including age, gender, culture, social class, and other identities may have an impact on the research process and outcomes [43]. Reflexivity on how the researchers’ position or views might have influenced the research design, process and findings is therefore essential. Several approaches were undertaken to ensure that the researchers did not have any influence on the research. An independent person recruited the participants. The researchers who conducted the interviews or those involved in the data analysis were not judgemental in any way. Further, an iterative process was followed in undertaking the data analysis (see above).

## 3. Results

The family and child characteristics of the study participants are presented in Table 2. Dental examination for children was conducted at 2 to 3 years of age. Of the children who completed their dental examination (*n* = 38), 76.3% were caries-free (decayed missing filled tooth surfaces (dmfs) = 0), while 23.7% had early childhood caries (dmfs = 1+). Data on child’s diet and toothbrushing practices were recorded at several timepoints. Data on sugar consumption were available for 41 children, of which 85.4% consumed sugary foods and drinks at least once a day. Data on toothbrushing frequency were available for 43 children, of which 53.5% brushed their teeth twice or more daily.

Five main themes emerged from the data. These included: (1) beliefs about child oral health and first set of teeth; (2) awareness and attitudes towards oral health services; (3) identification of caries risk and protective factors; (4) broader cultural and social class influences on childhood oral health practices; and (5) the influence of parental self-confidence, self-efficacy, and perceived control. The themes and sub-themes are presented in Table 3.

### 3.1. Theme 1: Beliefs about Child Oral Health and First Set of Teeth

There was a general lack of understanding regarding the role of a child’s baby teeth and the links between poor oral health in early childhood and later in life. Most mothers thought that the child’s first set of teeth were not important and the notion that they will “just fall out anyway” was shared amongst most of them. However, some mothers did agree that problems involving the primary dentition could somehow affect the eruption of adult teeth, but the answers were usually a guess (as this a qualitative study, the quotes of the participants are usually presented in italics.).

*“Yeah, because you can kind of think ‘well they lose these and move on to the real ones’, if you make a mistake, but I guess they’re important otherwise we wouldn’t have them. Not as important as the next lot but definitely not something to just ignore.”*

Almost all mothers did not associate the presence of dental caries in primary teeth with an increased risk of dental caries in permanent teeth. A more concerning attitude that was noted were responses from mothers who felt that the primary teeth acted as a “practice run” before the adult teeth came through. Only one mother made the link between learning good oral health behaviors in early childhood that could be carried over to adulthood. Additionally, most mothers had difficulty describing the functions of primary teeth other than for eating.

*“Well she’s got her first lot of teeth so if she’s got tooth decay now at least she’s got a second set coming but the set you have for life, that’s it.”*

Mothers recognized the importance of teeth in eating to provide good nutrition and the importance of adopting healthy behaviors early in life. Some mothers believed that good oral health included having good appearance of teeth that aided in self-confidence of the child.

*“I think he has better teeth than my daughter. His teeth are straight, not growing here and there… I gave my daughter a bottle till she was about 2 years and she got dental problems. Her front teeth were all rotten so she had them pulled out at the hospital. She was in pain and couldn’t eat. She wasn’t happy without front teeth as it looked awkward. With him, I am careful with what I feed him and now we all brush our teeth together as a family routine.”*

### 3.2. Theme 2: Awareness and Attitudes towards Oral Health Services

#### 3.2.1. Unrecognised Significance of Child’s First Dental Visit

Mothers had little knowledge regarding the recommended age for a child’s first dental visit and the frequency of visits thereafter. Some mothers had not even considered organizing a dental visit even after their child had all of their primary teeth erupted.

*“I haven’t heard anything, no, um… I haven’t really heard of kids having their teeth checked at this age so yeah I’m not too sure when you would have it done.”*

A few mothers defined the role of the dentist as more restorative as opposed to preventative. They recognized the need to take a child to a dentist if they had any dental problems, but did not consider a dental visit with the objective of assessment and monitoring or prevention. Some mothers did not understand how a dentist could help prevent dental problems.

*“I don’t really know how the dentist could prevent it. I usually go when I have pain.”*

#### 3.2.2. Limited Awareness on Seeking Oral Health Care

Most mothers did not know when to seek oral health-related advice. They did not think to look inside their child’s mouth to check their teeth and gums. Mothers that did look did not do so on a regular basis; and many did not know what they were looking for. Most mothers responded that they would know that their child had a dental problem if the child complained of pain or if they had problems feeding.

*“I think if he stopped eating, or something in his mouth, well he speaks so he could tell what happened. He would tell me what’s happening … that’s when I know there is some problem going!”*

Mothers reported that generally they were the decision-makers regarding the oral health of their children. However, some reported that healthcare decisions are made after discussions in the entire family, especially with grandparents of children. Additionally, many mothers relied on extended family and friends to recommend particular dentists.

*“My mother (grandmother) assists me to make dental appointments and choose dentists for them (children).”*

Most mothers were unaware of the free oral health services available to children. They were surprised that children could have free dental appointments without any personal costs, and believed this was crucial to raise the awareness of oral health.

*“I am quite surprised…. I did not even know all my children could get free check-ups through the government. I have been paying for my other kids all this while. I am pretty sure all my friends don’t know this as well. I think the government should raise more awareness about this as people are unaware that they can get free dental services for children.”*

#### 3.2.3. Past Dental Experiences and Necessity of Trust in Oral Health Professionals

Some mothers reported that they avoided regular attendance to oral health care professionals unless their child was experiencing pain as dental treatment was often unpleasant. Reasons for avoidance included bad prior experiences and fear related to experiences with dental procedures such as dental restorations and tooth extractions. The environment of the clinic, in particular the sounds of drill, the sight of local anaesthesia instruments, and the smell of some materials were common reasons for avoidance.

*“She had to get one of her tooth pulled out as she was in pain. It was a bad experience as she was in pain. She hates going to the dentist now as she is scared of injections.”*

A few mothers reported that the communication and trust between the oral health professional and patient is really important in preventing and managing oral health.

*“My dentist is excellent… I have known her for 10 years. She will make things so simple and clear for me. That way, I know what is wrong and what I need to do.”*

Some mothers felt that they received questionable treatment themselves at a few dentists, and perceived that some clinicians in private practice performed unnecessary treatment to earn more money. Those mothers did not feel comfortable bringing their child to the same environment as they had lost trust in these private practitioners.

*“My previous dentist told me I had 3 cavities that needed filling. I got a bit worried as I go for regular check-ups, so I saw another dentist.”*

Differences in private and public oral health care was reported by some mothers who related their experiences and personal circumstances in deciding which service to attend. In particular, mothers who attended public dental services were satisfied with the care provided by oral health professionals.

*“I am on low income, so I can’t afford going to a private dentist. I go to the local community health clinic. There is a waiting list, but the staff there are excellent. The lady (oral health therapist) that treats him (my son) is so good with him. He feels so comfortable around her.”*

### 3.3. Theme 3: Identification of Caries Risk and Protective Factors

Several caries risk and protective factors were identified by the mothers.

#### 3.3.1. Child’s Diet

Mothers reported children having preferences for certain foods or being “fussy eaters” as barriers to maintaining a healthy diet. Most mothers considered sugar to be harmful for teeth, yet reported that they have difficulty in limiting their child’s sugar consumption, predominantly due to child temperament.

*“Sometimes when he goes to the supermarket, he will get a tantrum… I feel embarrassed so I have to buy him some lollies or else he keeps nagging.”*

Most children snacked on healthy items such as fruit and yoghurt. Some reported having unhealthy snack items such as chips and chocolate. Some mothers were surprised that savory snacking items had sugar.

*“She’ll either have a piece of fruit or chips just as a snack…. I wouldn’t have thought chips had sugar…”*

Most mothers were unaware of the hidden sugars in certain food items. They reported that they did not check food labels when purchasing food and drink items.

*“Oh Dear! I didn’t know a small bottle of soft drink had 10 teaspoons of sugar. I thought it would have a few but not 10. I never read the labels.”*

Most mothers gave water to their children with meals and something special such as fruit juices and soft drinks in between meals. Fruit juices were mainly used as a treat and were usually diluted with water. While mothers knew that the sugar component of fruit juice was not good for their child’s teeth, they did not take into consideration the frequency with which sugar and acid was given to their child. Apart from diluting the juice, water was not regularly given to the child after drinking the juice or soft drink to wash the acid/sugar away.

*“Probably once a day… lemonade with water, diluted, we always dilute it.”*

#### 3.3.2. Sharing of Spoons and Dummies

The majority of mothers did share spoons with their children and did not consider the possibility of any transfer of bacteria. On questioning further, mothers reported that they symbolized this as a form of sharing love within the family.

*“We all share… they’ll eat something that I’ve got because they want it, so yeah we do share food um… not just spoons.”*

Mothers reported that the use of a dummy was a common practice in infants. Some mothers reported that they would lick the dummy themselves if the dummy fell on the floor and then give it to the child. Interestingly, they believed that bacteria from the floor would be transferred to themselves, but never thought of the possibility of transferring their bacteria to their child.

*“If her dummy falls on the floor, I will put it in my mouth first before I give it to her. That way, I get the germs… not her…”*

#### 3.3.3. Fluoride Use and Tooth Brushing Practices

In general, mothers acknowledged the importance of twice daily toothbrushing with a fluoride toothpaste to maintain good oral health for their children. However, some were scared that young children might swallow a large amount of toothpaste, and thus avoided its use until they felt that the child was ready.

*“She is too young to brush her teeth with a toothpaste. She will swallow it because of the strawberry flavour.”*

Most mothers were aware of the benefits of fluoride in preventing tooth decay. Interestingly, some mothers were aware of fluoride either through television advertisements or by the advice of their oral health practitioner.

*“It is the fluoride in the toothpaste that gives the protection…. That’s what they show in all the TV advertisements.”*

### 3.4. Theme 4: Broader Cultural and Social Class Influences on Childhood Oral Health Practices

#### 3.4.1. Cultural Influences

Some mothers reported that the cultural background of the family strongly influences the beliefs, attitudes, and health behaviors of children. In particular, a few mothers reported that some traditional beliefs in Eastern culture were very different from Western culture. Mothers had different opinions about tap water which were influenced by their own cultural and childhood experiences.

*“In Australia, I grew up drinking tap water and I’ve passed that on to my children as well”*

*“You see…drinking tap water is a bit ‘western’ style, not a ‘Chinese’ way.”*

Some mothers had beliefs that were not conducive to promoting healthy behaviors in early childhood. These primarily related to diet and some mothers’ beliefs that sweets were acceptable for children because they burn off the calories.

*“Sweets between the meals gives him energy. We do that all the time in our home country, Lebanon.”*

Whilst most mothers reported that seeking medical help in early childhood was important, some mothers believed that oral health care was something separate and should be accessed when there was an obvious problem. The concept of routine preventative visits to oral health professionals was not clearly articulated amongst a few culturally diverse mothers. These mothers expressed the opinion that their personal preventative practices would make the visit to the oral health professional unnecessary.

*“In China, we believe that if parents can take good care of their child’s teeth, there is no need to see a dentist, but they should see a dentist once there is a problem.”*

Stories about traditional oral health practices were revealed in some interviews conducted with mothers from cultures outside of Australia. The use of “miswak” and “neem” were a common practice in Lebanese and Indian families, respectively. Mothers used these traditional practices themselves for a “fresh” feeling and planned to introduce them to their children. There was a common perception that these traditional items had anti-bacterial properties and would help protect them from oral health problems.

*“It is not for religious reasons, it (miswak) is proven to clean your teeth. It is used in many cultures now including Lebanese.”*

*“I see Indian videos all the time that it (neem) kills the germs and is good for a fresh breath.”*

#### 3.4.2. Relative Social Standing

A few families in this study had low incomes and strongly believed that income played a major role in developing health promoting behaviors. Some mothers believed that relative social standing in their community strongly influenced their child’s oral health. The general agreement was that families that were rich could afford healthier foods and visits to the dentist. Interestingly, they were unaware that child oral health services were free and easily accessible.

*“I am not on high wages. How am I supposed to pay the dentist if I get paid so little? What do you want me to do?”*

Some mothers did not believe that diet and twice daily toothbrushing efforts could fully prevent their child from dental caries, defined as an external locus of control. A few mothers believed that their initiatives or behaviors would not lead to prevention of dental caries or make any difference. They often related dental caries to causes outside the parent’s and child’s control. Mothers perceived factors outside one’s control, such as luck, chance, genetics, and lower socioeconomic status to be responsible for the occurrence of dental caries.

*“I get upset and angry that we have to deal with all this because we are not rich. I am just born with bad luck and genetics, for which I can’t do anything about.”*

*“Our child is likely to get dental caries no matter what we do. I don’t think brushing everyday would make any difference. Sometimes it is just by chance.”*

### 3.5. Theme 5: The Influence of Parental Self-Confidence, Self-Efficacy and Perceived Control

#### 3.5.1. Parental Self-Confidence and Self-Efficacy

Most mothers conveyed that they had the belief and confidence in themselves that they will adopt healthy behaviors for their children, such as toothbrushing with a fluoride toothpaste. Some expressed self-confidence in their ability to successfully establish and maintain a twice daily toothbrushing routine for their child, which indicated a high self-efficacy. Parental self-efficacy was observed, particularly with some mothers expressing confidence on the positive outcomes of their parenting role on their child’s oral health behaviors.

*“I make sure that the task of twice daily toothbrushing is completed every day.”*

*“I believe that by reminding my kid to brush twice a day, I am making a difference to their oral health.”*

Most mothers managed to brush their child’s teeth twice a day, usually in the morning before or after breakfast and in the evening before bedtime. However, a few expressed that their child insisted on being able to brush their teeth themselves and did not allow the mothers to do it for them. Some parents reported non-compliance as a barrier to toothbrushing due to difficult behaviors such as tantrums and mentioned that they did not want to escalate such situations. Mothers shed light on several strategies they adopted to ensure toothbrushing was fun and a part of regular lifestyle behavior. These included providing rewards, such as stickers, colouring charts, or rewarding words, using a timer, counting along, and showing cartoon videos on toothbrushing.

*“He wants to play with his toothbrush in the morning. I wouldn’t sit and argue with him in the morning to get late to work. I just lure him with stickers or something he likes, so we make toothbrushing a fun activity for his daily morning routine.”*

Parental supervision of toothbrushing was seen as important as children lacked manual dexterity and brushing on their own would be ineffective. Most parents also indicated role modeling or setting an example for the child by brushing their own teeth in the presence of their children. However, a few expressed difficulties due to time constraints, especially in the morning. Some mothers mentioned that they wanted to encourage the child and build his/her confidence and not make them feel that they could not do it themselves.

*“I let him play with his brush first, so he can feel that he can do it himself. I will then go over his brushing to ensure he has done it correctly.”*

#### 3.5.2. Perceived Parental Control

Although a sugary diet was identified as causing oral health problems, mothers felt they had little control over what their children were eating. In particular, this was a concern as their children grew older, as mothers felt they were not able to monitor their child’s diet, and felt parents were often blamed when their child had health problems. There was a sense of helplessness that children do not listen to their parents, and this concerned them. Mothers reported that raising children in Australia is very different from how they were raised, and this worried them greatly.

*“When I was a child, I used to listen to my parents, and we had a healthy diet and play time. These days children sit and watch TV, eat what they want, and don’t listen to their parents anymore.”*

Most mothers expressed the importance of promoting healthy eating behaviors. Some mothers reported that they had a good control of foods and drinks at home as they had set house rules regarding sugary foods.

*“If he is really hungry, I will ask him to snack on a piece of fruit. I don’t buy chocolates and sugary drinks… If they (chocolates and sugary drinks) are not at home, he won’t ask… He is only allowed to eat them as a treat once in a while…”*

Most mothers believed that they had little control over their child’s diet when children were outdoors. A few expressed concerns about the influence of family and friends, and supermarket and television commercials advertising on their child’s diet and oral health. Some parents reported that children often ate sugary foods and drinks when visiting neighbors, friends and relatives (particularly grandparents), and local shops.

*“Grandparents are the worse! I get into arguments as they (grandparents) buy them (children) lollies all the time.”*

## 4. Discussion

This study provides useful insights into perceptions and understanding of mothers residing in GWS about various factors that influence the early childhood oral health. Mothers reported having limited knowledge and awareness on the importance of primary teeth, a child’s first dental visit, and oral health care. Mothers also expressed the necessity of trust in oral health professionals and identified several caries risk and protective factors. They also highlighted the influence of culture and social class, and parental self-confidence, self-efficacy, and perceived control on child oral health.

Mothers had limited knowledge on the importance of primary teeth and considered them to be temporary and not as vital as permanent teeth. Several studies [26,44] have reported similar findings indicating that the low importance was attributed to parental belief that primary teeth would shed off giving way to permanent teeth. Consistent with the views reported in other similar studies [22,26], most mothers were unaware of the association between caries experience in primary teeth and caries incidence in permanent teeth. This is of concern as research suggests that children with caries in their primary teeth are three times more likely to develop caries in their permanent teeth [7]. These preconceived beliefs held by mothers about child oral health presents a grave challenge to developing effective intervention programs for oral health promotion [45].

In addition to some mothers not being aware of the recommended timing for their child’s dental visit, it was concerning to observe that some mothers were unaware of the role of oral health professional in early prevention and intervention. This finding is consistent with a study conducted in Western Australia [46] where only 12% of children aged 2 years and below were taken for a dental visit. The Australian Institute of Health and Welfare also reported that only 28% of children aged 2 to 4 years visited the dentist in the last 12 months [47]. On the contrary, infants were reported to be taken to primary healthcare services 35 times on average in their first year of life, none of which were visits to a dental professional [48]. This study also highlighted that there was a general lack of maternal knowledge about effectively identifying and seeking help for preventing their child’s oral health problems. Although some mothers were keen to identify oral health issues by checking their child’s teeth and gums, they did not know what to look for, which could be attributed to the lack of emphasis on prevention-based dental care and poor care-coordination [49]. Moreover, mothers were unaware of the public dental services that provides treatment free of cost to children under 18 years of age. One of the main reasons contributing to this issue could be the increased trend of privatization of health services which has not only impacted the affordability of healthcare, but also undermined the use of public health services [50]. On a more positive note, mothers taking their child for dental visits as recommended expressed the importance of trust in oral health professionals, as reported in a previous systematic review [51].

Mothers were successful in identifying various caries risk and protective factors. Mothers had a better perception on the role of diet in their child’s dental health as compared to other factors. There was a general consensus among mothers that sugar was harmful for teeth. However, most mothers were not aware of the sugar content in most snacking items and admitted their failure to check the sugar content in food labels as noticed in other studies [14,52]. Sharing of spoons and dummies was another issue that was reported among many mothers as they were not aware of the risk of tooth decay associated with transfer of cariogenic bacteria to their children. Several studies have consistently shown that caries could be transmitted from a mother to her child through the sharing of food and eating utensils [53,54]. On the contrary, the majority of mothers were familiar with the importance of toothbrushing with a fluoride toothpaste. A positive influence of advertisements on child oral health was also observed with some mothers expressing that they learnt about the benefits of fluoride from television advertisements.

Perceptions towards health as a result of broader cultural influences are a relatively common issue especially in the multi-cultural population [55]. As opposed to Australian-born mothers, Asian mothers did not prefer to drink tap water. Some Middle Eastern mothers believed that providing sweets between meals acted as a source of energy for their children. Whilst not all cultural practices have negative influence, it is important for health professional teams to identify and educate mothers in a culturally appropriate manner to avoid practices that could potentially affect their child’s oral health [56]. Alternatively, the influence of relative social standing on child oral health was also emphasized by some mothers. Those with a low income further conveyed their belief that only high-income families can afford healthy food and dental visits. Cost of dental treatment has often been reported as one of the known barriers to regular utilization of dental services [9]. This study also presented an intriguing finding that some mothers believed that oral health measures would not fully prevent dental caries and associated these with factors outside their control such as luck and lower socio-economic status. Previous studies have reported similar findings on parental locus of control [14,24]. Moreover, mothers with more external locus of control have been found to have children with greater caries risk [57]. Research also suggests that lower income and educational attainment are associated with higher external locus of control which could lead to worse oral health outcomes [58].

Most mothers were confident of achieving twice daily toothbrushing in their children and some used playful strategies. Previous studies suggested that parental self-efficacy plays an essential role in child oral health, particularly in instilling toothbrushing habits in children [59,60]. It is essential to utilize a family-based approach and develop dental caries prevention interventions aimed at improving parental dental self-efficacy and parental skills in positive reinforcement and habit formation [14]. Some mothers were confident that they had good control over their child’s sugary food consumption. Others reported that their choice for healthy eating were most often compromised by their child’s preference for certain foods and influence of family or friends, as reported in other similar studies [14,61]. Some mothers also expressed concern that they did not have control over their child’s diet as children in Australia rarely listen to their parents. Difficulties in raising children in Australia as a migrant and its influence on child oral health have also been discussed in a previous study [24]. It is imperative that these parental factors are taken into account when developing interventions for promoting child oral health.

### 4.1. Strengths and Limitations

This study has several strengths worth reporting. Firstly, a qualitative research design was used to gain an in-depth understanding of the views of the mothers residing in GWS. The flexibility of the study design allowed for simultaneous data collection and analysis and provided an opportunity for further investigation [36]. Secondly, this qualitative study was nested within the HSHK study, and all mothers (*n* = 45) invited to take part in the study consented, hence achieving a response rate of 100%. Thirdly, the sample size of 45 mothers was adequate to reach data saturation, which indicates that all the dimensions of interest were explored and no new information would have been collected from interviewing new participants [36].

The study findings should be considered in the light of some limitations. Firstly, the generalizability of the study is limited by the use of a small sample of mothers residing in GWS. Secondly, although several approaches were undertaken with regards to researcher positionality, the researchers’ position might have influenced the research findings and mothers may have responded in a socially desirable way. Thirdly, only mothers’ perspectives were explored as they are often the primary carers of young children. However, other family members may have different perspectives. Future research should be aimed towards exploring and comparing the perspectives of other family members with that of mothers regarding the factors that influence child oral health to overcome any barrier to child oral health in socio-economically disadvantaged communities.

### 4.2. Implications and Recommendations

Based on the mother’s perceptions and understanding, several barriers and facilitators to maintaining early childhood oral health were identified. Barriers included limited knowledge on the importance of primary teeth, child’s first dental visit, and seeking oral health care; little control over child’s diet including sugar consumption; sharing of spoons and dummies; and some cultural and social class influences. Alternatively, facilitators included communication and trust in oral health professionals; awareness of the importance of twice daily toothbrushing with a fluoride toothpaste; parental self-confidence and self-efficacy; and parental control on child’s diet. These findings may help inform the development or improvement of oral health promotion interventions.

When designing health promotion programs and resources targeted at disadvantaged and socio-economically deprived communities, engagement of the end users is essential. In order to ensure that research is translated into practice, researchers and policy makers should also engage consumers in all phases [62]. Community engagement is also a vital tool to address health inequality as it empowers and encourages those from disadvantaged communities to take control of their own health [63]. Oral health promotion initiatives should also focus at empowering and educating mothers so that they gain confidence and control over the oral health of their families. Similarly, oral health promotion education and interventions should be embedded early on and the oral health resources disseminated to families through early childhood day care centers and preschools. The need for a shared model of care integrating interprofessional collaboration of child and family health nurses, paediatricians, general practitioners, and oral health professionals was also identified as most mothers were unaware of the importance of seeking dental visits in the early childhood. Using a model of shared responsibility allows for early assessment and intervention; it helps to identify children with or at risk of developing dental caries, so that they can be referred to oral health care services [64].

Oral health education is an essential component of oral health promotion, therefore theory-based educational interventions such as the theory of planned behavior (TBP), which are often successful in maintaining healthy behaviors are recommended [65]. The TBP illustrates the key elements of healthy behavior and comprises five constructs—intention, attitude, subjective norm, and perceived behavioral control [66]. Education intervention programs based on TBP may help improve mothers’ attitude, intention, and perceived control towards oral health, and their children’s oral health behavior [65,67]. Future research could also explore the findings on mothers with internal locus of control, self-efficacy, and perceived parental control further with the application of a behavioral model as TBP to understand its influence on behaviors such as toothbrushing.

## 5. Conclusions

This study provides valuable insights and in-depth understanding on the perceptions and understanding of mothers residing in the GWS, one of Australia’s most culturally diverse and socio-economically disadvantaged regions, on the factors that influence early childhood oral health. Mothers reported having limited knowledge on the importance of the first set of teeth. Mothers’ awareness and attitudes towards oral health services revealed unrecognized significance of child’s first dental visit, limited awareness on seeking oral health care, influences of past dental experiences on child oral health, and the necessity of trust in oral health professionals. Several caries risk and protective factors such as child’s diet, sharing of spoons and dummies, and fluoride and tooth brushing practices were identified by the mothers. Mothers highlighted broader cultural and social class influences on childhood oral health practices. Moreover, the influence of parental self-confidence, self-efficacy, and perceived control on child oral health were observed. The empirical findings of this qualitative study bring our attention to the critical factors that influence child oral health and the opportunities for child oral health promotion by targeting mothers.

## Figures and Tables

**Table 1:** Semi-structured interview guide.

Topics of Discussion for the Interview
1. How do you feel about your child’s oral health?
2. Where do you get all the information you know about how to look after your child’s teeth?
3. What do you think helps in maintaining good oral health for your child?
4. What do you think is bad for your child’s teeth?
5. How do parents influence their child’s oral health?
6. What other factors do you believe influence children’s oral health?

**Table 2:** Family and child characteristics of the study participants (*n* = 45).

Characteristic	*n*
**Family Characteristics**	
Age (in years)	
20–24	8
25–29	12
30–34	11
35–39	8
40+	6
Parity	
One child	17
More than 1 child	28
Education level of the mother	
≤Year 12	14
Some Tertiary education	16
Completed University education	15
Marital status of the mother	
Living with partner	42
Single	3
Mother’s country of birth	
Australia	17
China	6
Lebanon	7
India	9
Vietnam	6
Annual family income (Australian Dollars)	
<50 k	14
50 k–100 k	21
>100 k	10
**Child Characteristics**	
Child’s gender	
Male	24
Female	21
decayed missing filled tooth surfaces (dmfs) (*n* = 38)	
0	29
1	1
2	2
3+	6
Child’s sugar consumption (*n* = 41)	
At least once a day	35
Less than once a day	6
Child’s toothbrushing frequency (*n* = 43)	
Twice or more daily	23
Less than twice daily	20

**Table 3:** Summary of themes, subthemes, and examples.

Themes	Subthemes	Examples
Theme 1: Beliefs about child oral health and first set of teeth		General lack of understanding on: Importance of a child’s primary teeth Links between poor oral health in early childhood and adulthood
Theme 2: Awareness and attitudes towards oral health services	Unrecognised significance of child’s first dental visit	Limited knowledge on: Recommended age for child’s first dental visit Importance of dental visits for prevention
	2. Limited awareness on seeking oral health care	Lack of awareness on: When to seek oral health related advice Free oral health services available to children
	3. Past dental experiences and necessity of trust in oral health professionals	Avoidance of regular attendance to oral health professionals due to prior bad experiences Importance of communication and trust
Theme 3: Identification of caries risk and protective factors	Child’s diet	Difficulty in reducing sugar consumption Unawareness of sugars in certain food items Failure to check food labels before purchase
	2. Sharing of spoons and dummies	Sharing spoons and licking dummies Did not consider the possibility of transfer of bacteria to their child
	3. Fluoride and tooth brushing practices	Acknowledged the importance of twice daily toothbrushing with a fluoride toothpaste Avoidance of toothpaste until the child is ready
Theme 4: Broader cultural and social class influences on childhood oral health practices	Cultural influences	Influence of cultural background on beliefs, attitudes, and health behaviors of children Some beliefs were not conducive to promoting healthy behaviors in early childhood
	2. Relative social standing	Strong influence of relative social standing and lower income on child’s oral health External locus of control- relating dental caries to causes outside one’s control
Theme 5: The influence of parental self-confidence, self-efficacy and perceived control	1. Parental self-confidence and self-efficacy	Self-confidence in ability to successfully establish a toothbrushing routine for their child Influence of self-efficacy on health behaviors Importance of supervision of toothbrushing
	2. Perceived parental control	Good control over sugary foods at home Little control over child’s diet outdoors

## Data Availability

The data presented in this study are not publicly available due to privacy.

## Supplementary Materials

The following are available online at https://www.mdpi.com/article/10.3390/ijerph18073521/s1, Table S1: COREQ (COnsolidated criteria for REporting Qualitative research) Checklist, Figure S1: Thematic map showing the interaction between themes and subthemes.
