# Supplementary material for: How Do Mothers Living in Socially Deprived Communities Perceive Oral Health of Young Children? A Qualitative Study
Source: Int J Environ Res Public Health. 2021 Mar 29;18(7):3521. doi: 10.3390/ijerph18073521 (PMC8038038; doi:10.3390/ijerph18073521)
Supplement: Supplementary file 1 [file ijerph-18-03521-s001.zip › ijerph-1098218-supplementary.docx]

**Supplementary Material**

**Table S1.** COREQ (COnsolidated criteria for REporting Qualitative research) Checklist.

| **Item No. and Topic** | **Guide Questions/Description** | **Response and Reported on Page No.** |
| --- | --- | --- |
| **Domain 1: Research team and reﬂexivity** | | |
| *Personal characteristics* | | |
| 1. Interviewer/facilitator | Which author/s conducted the interview or focus group? | D.L., M.T. and A.A. (Page 4) |
| 2. Credentials | What were the researcher’s credentials? E.g. PhD, MD | A.A.: MPH, PhD, Senior Lecturer  D.L.: BDent  M.T.: BDent  R.C.: MPH, PhD Candidate  S.B.: MDSc, Clinical Professor  S.K.T.: MDSc, PhD, Senior Research Fellow  J.J.C: MS, ScD, Professor |
| 3. Occupation | What was their occupation at the time of the study? | Research students and researchers |
| 4. Gender | Was the researcher male or female? | All male |
| 5. Experience and  training | What experience or training did the researcher have? | Interviewers had prior experience in qualitative interviewing and population oral health (Page 5). Other researchers are involved in other research projects. |
| *Relationship with participants* | | |
| 6. Relationship  established | Was a relationship established prior to study commencement? | A.A. had recruited the participants for birth cohort sudy on child oral health. Other researchers had no relationships with the participants before the study. |
| 7. Participant knowledge  of the interviewer | What did the participants know about the researcher? e.g. personal goals, reasons for doing the research | Participants knew about the reasons for doing research as indicated in the participant information statement (Page 4). |
| 8. Interviewer  characteristics | What characteristics were reported about the inter viewer/facilitator? e.g. Bias, assumptions, reasons and interests in the research topic | Apart from their names and affiliation, participants did not know about interviewers’ characteristics. |
| **Domain 2: Study design** | | |
| *Theoretical framework* | | |
| 9. Methodological  orientation and Theory | What methodological orientation was stated to underpin the study? e.g. grounded theory, discourse analysis, ethnography, phenomenology, content analysis | Qualitative Research Design and Fisher-Owens Model (Page 3) |
| *Participant selection* | | |
| 10. Sampling | How were participants selected? e.g. purposive, convenience, consecutive, snowball | Purposive (Page 3) |
| 11. Method of approach | How were participants approached? e.g. face-to-face, telephone, mail, email | Telephone (Page 4) |
| 12. Sample size | How many participants were in the study? | 45 mothers (Page 4) |
| 13. Non-participation | How many people refused to participate or dropped out? Reasons? | None refused to participate or dropped out |
| *Setting* | | |
| 14. Setting of data  collection | Where was the data collected? e.g. home, clinic, workplace | Home interview (Page 4) |
| 15. Presence of non-  participants | Was anyone else present besides the participants and researchers? | Only the participants and interviewers were present (Page 4) |
| 16. Description of  sample | What are the important characteristics of the sample? e.g. demographic data, date | Family and child characteristics of the study participants are outlined in Table 2 (Page 6) |
| *Data collection* | | |
| 17. Interview guide | Were questions, prompts, guides provided by the authors? Was it pilot tested? | Semi-structured interview guide is presented in Table 1. It was also pilot tested (Page 4). |
| 18. Repeat interviews | Were repeat interviews carried out? If yes, how many? | Repeat interviews were not carried out |
| 19. Audio/visual  recording | Did the research use audio or visual recording to collect the data? | Interviews were audio-recorded (Page 4) |
| 20. Field notes | Were ﬁeld notes made during and/or after the interview or focus group? | Field notes were made immediately after each interview and they helped in data analysis. |
| 21. Duration | What was the duration of the interviews or focus group? | One hour (Page 4) |
| 22. Data saturation | Was data saturation discussed? | Yes (Page 4 and 5) |
| 23. Transcripts returned | Were transcripts returned to participants for comment and/or correction? | Yes (Page 5) |
| **Domain 3: Analysis and ﬁndings** | | |
| *Data analysis* | | |
| 24. Number of data  coders | How many data coders coded the data? | Four researchers- A.A., M.T., D.L. and R.C. (Page 5) |
| 25. Description of the  coding tree | Did authors provide a description of the coding tree? | Yes (Figure S1) |
| 26. Derivation of themes | Were themes identiﬁed in advance or derived from the data? | Themes were derived from the data (Page 5) |
| 27. Software | What software, if applicable, was used to manage the data? | NVivo 9 software (Page 5) |
| 28. Participant checking | Did participants provide feedback on the ﬁndings? | Yes |
| *Reporting* | | |
| 29. Quotations presented | Were participant quotations presented to illustrate the themes/ﬁndings? Was each quotation identiﬁed? e.g. participant number | Yes, participant quotations were presented (Page 7-12). Quotations were de-identified for confidentiality reasons. |
| 30. Data and ﬁndings  consistent | Was there consistency between the data presented and the ﬁndings? | Yes, as indicated in Rigor (Page 5) |
| 31. Clarity of major  themes | Were major themes clearly presented in the ﬁndings? | Yes, presented in Results (Page 7-12) |
| 32. Clarity of minor  themes | Is there a description of diverse cases or discussion of minor themes? | Yes, presented in Discussion (Page 13-14) |

Developed from: Tong A, Sainsbury P, Craig J. Consolidated criteria for reporting qualitative research (COREQ): a 32-item checklist for interviews and focus groups. *International Journal for Quality in Health Care*. 2007. Volume 19, Number 6: pp. 349—357.

**
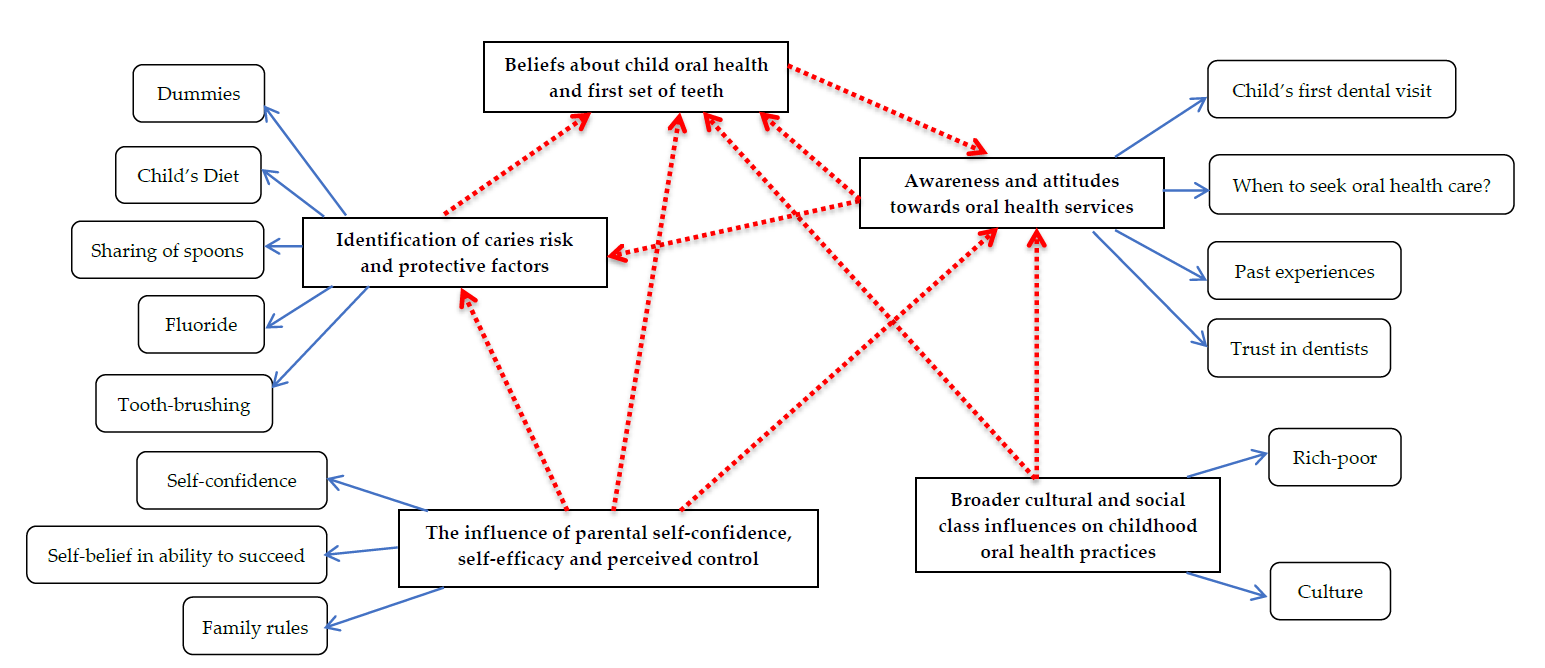
**

**Figure S1.** Thematic map showing the interaction between themes and subthemes.
